# Supplementary material for: Association between Pre-Existing Long-Term β-Blocker Therapy and the Outcomes of Sepsis-Associated Coagulopathy: A Retrospective Study
Source: Medicina (Kaunas). 2022 Dec 15;58(12):1843. doi: 10.3390/medicina58121843 (PMC9786011; doi:10.3390/medicina58121843)
Supplement: Supplementary file 1 [file medicina-58-01843-s001.zip › medicina-2062678-supplementary.pdf]

Association between pre-existing long-term β-blocker therapy and the outcomes of sepsis-associated coagulopathy: A retrospective study

Ying Ma [1], Jie Ma [2] and Jiong Yang [3,*]

[1] Department of Respiratory and Critical Care Medicine, Zhongnan Hospital of Wuhan University, Wuhan 430071, China; mayingwhu@126.com
[2] Department of Mathematics and Physics, North China Electric Power University–Baoding, Baoding 071003, China; majie201407@126.com
[3] Department of Respiratory and Critical Care Medicine, Zhongnan Hospital of Wuhan University, Wuhan 430071, China; yjiongwhu@126.com
* Correspondence: yjiongwhu@126.com; Tel.: +86-027-67813277

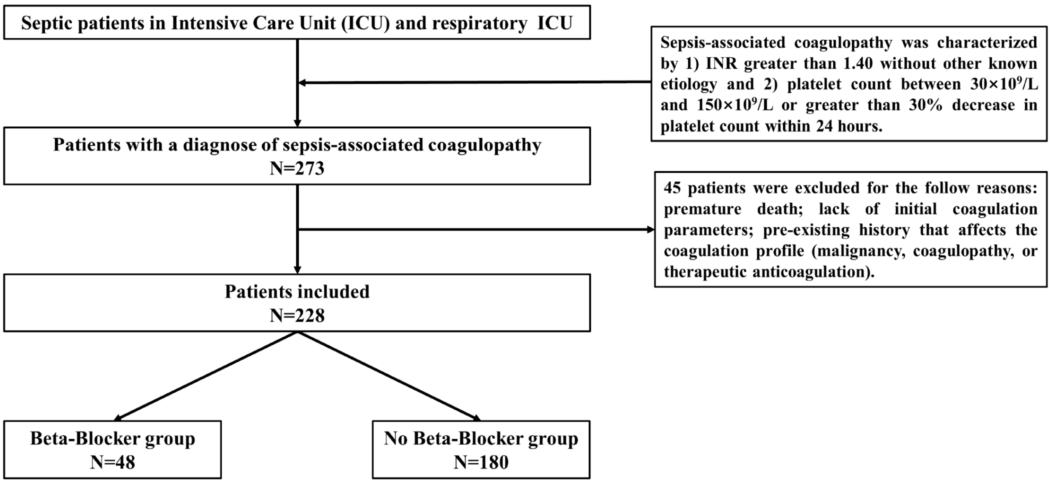

Figure S1. Flow diagram of the cohort study.
